# Exometabolomic Analysis of Decidualizing Human Endometrial Stromal and Perivascular Cells

**DOI:** 10.3389/fcell.2021.626619

**Published:** 2021-01-28

**Authors:** Sarah L. Harden, Jieliang Zhou, Seley Gharanei, Maria Diniz-da-Costa, Emma S. Lucas, Liang Cui, Keisuke Murakami, Jinling Fang, Qingfeng Chen, Jan J. Brosens, Yie Hou Lee

**Affiliations:** ^1^Division of Biomedical Sciences, Clinical Science Research Laboratories, Warwick Medical School, University of Warwick, Coventry, United Kingdom; ^2^Singapore–MIT Alliance for Research and Technology, Singapore, Singapore; ^3^Institute of Molecular and Cell Biology, Agency for Science, Technology and Research, Singapore, Singapore; ^4^Translational ‘Omics and Biomarkers Group, KK Research Centre, KK Women’s and Children’s Hospital, Singapore, Singapore; ^5^Warwickshire Institute for the Study of Diabetes, Endocrinology and Metabolism (WISDEM), University Hospitals Coventry and Warwickshire NHS Trust, Coventry, United Kingdom; ^6^Tommy’s National Centre for Miscarriage Research, University Hospitals Coventry and Warwickshire, Coventry, United Kingdom; ^7^Centre for Early Life, Warwick Medical School, University of Warwick, Coventry, United Kingdom; ^8^Department of Obstetrics and Gynecology, Faculty of Medicine, Juntendo University, Tokyo, Japan; ^9^Obstetrics and Gynaecology Academic Clinical Programme, Duke-NUS Medical School, Singapore, Singapore

**Keywords:** endometrium, decidualization, exometabolome, metabolism, perivascular cells, reproduction, endometrial stromal cells

## Abstract

Differentiation of endometrial fibroblasts into specialized decidual cells controls embryo implantation and transforms the cycling endometrium into a semi-permanent, immune-protective matrix that accommodates the placenta throughout pregnancy. This process starts during the midluteal phase of the menstrual cycle with decidual transformation of perivascular cells (PVC) surrounding the terminal spiral arterioles and endometrial stromal cells (EnSC) underlying the luminal epithelium. Decidualization involves extensive cellular reprogramming and acquisition of a secretory phenotype, essential for coordinated placental trophoblast invasion. Secreted metabolites are an emerging class of signaling molecules, collectively known as the exometabolome. Here, we used liquid chromatography-mass spectrometry to characterize and analyze time-resolved changes in metabolite secretion (exometabolome) of primary PVC and EnSC decidualized over 8 days. PVC were isolated using positive selection of the cell surface marker SUSD2. We identified 79 annotated metabolites differentially secreted upon decidualization, including prostaglandin, sphingolipid, and hyaluronic acid metabolites. Secreted metabolites encompassed 21 metabolic pathways, most prominently glycerolipid and pyrimidine metabolism. Although temporal exometabolome changes were comparable between decidualizing PVC and EnSC, 32 metabolites were differentially secreted across the decidualization time-course. Further, targeted metabolomics demonstrated significant differences in secretion of purine pathway metabolites between decidualized PVC and EnSC. Taken together, our findings indicate that the metabolic footprints generated by different decidual subpopulations encode spatiotemporal information that may be important for optimal embryo implantation.

## Introduction

Cyclical decidualization, i.e., differentiation of endometrial fibroblasts into specialized decidual cells, is a hallmark of menstruating mammals (Brosens et al., 2009; Emera et al., 2012). Rather than being triggered by an implanting embryo, decidualization in menstruating species is initiated during the midluteal phase of each cycle in response to sustained progesterone signaling and rising intracellular cyclic adenosine monophosphate (cAMP) levels (Gellersen and Brosens, 2014). Initially, decidual changes are most prominent in sushi domain-containing 2-positive (SUSD2+) cells surrounding the terminal spiral arterioles and in SUSD2− stromal cells underlying the luminal epithelium (Murakami et al., 2014), and then spread to encompass the entire stromal compartment (Gellersen and Brosens, 2014). In parallel, proliferating innate immune cells accumulate, foremost uterine natural killer cells (Brighton et al., 2017). Upon embryo implantation, decidualizing cells rapidly encapsulate the conceptus (Weimar et al., 2012; Berkhout et al., 2018), engage in embryo biosensing and selection (Brosens et al., 2014; Macklon and Brosens, 2014), and then form an immune-privileged decidual matrix that controls interstitial and intravascular trophoblast invasion (Nancy et al., 2012; Erlebacher, 2013; Gellersen and Brosens, 2014).

Recent studies have shown that decidualization is a multistep differentiation process, which starts with an evolutionarily conserved acute cellular stress response (Erkenbrack et al., 2018; Stadtmauer and Wagner, 2020), characterized by a burst of reactive oxygen species (ROS) production and release of proinflammatory cytokines (Al-Sabbagh et al., 2011; Salker et al., 2012; Brighton et al., 2017; Lucas et al., 2020). After a lag period of several days, differentiating cells lose their fibroblastic appearance and emerge as secretory decidual cells with abundant cytoplasm and prominent endoplasmic reticulum (Gellersen and Brosens, 2014). At a molecular level, the decidual transformation of endometrial fibroblasts involves genome-wide remodeling of the chromatin landscape (Vrljicak et al., 2018) extensive reprogramming of multiple signal transduction and metabolic pathways (Leitao et al., 2010; Muter et al., 2016, 2018) and activation of decidual gene networks (Takano et al., 2007; Cloke et al., 2008; Mazur et al., 2015). Decidualization of EnSC has been associated with alterations in respiration and mitochondrial metabolism (Ryu et al., 2020). At a functional level, decidualization transforms endometrial fibroblasts into secretory cells that are anti-inflammatory (Kuroda et al., 2013; Lucas et al., 2020), resistant to stress signals (Kajihara et al., 2006; Leitao et al., 2010; Lucas et al., 2020), and highly responsive to embryonic cues (Teklenburg et al., 2010; Brosens et al., 2014).

The decidual secretome is highly dynamic. Its composition changes across the different phases of the decidual pathway (Salker et al., 2012; Lucas et al., 2016, 2020) and the amplitude of the secretory response depends on the topography of cells in the native tissue (Murakami et al., 2014). For example, analysis of paired SUSD2− and SUSD2+ cell cultures demonstrated that perivascular SUSD2+ cells secrete significantly higher levels of various chemokines and cytokines, including chemokine (C-C motif) ligand 7 (CCL7) and leukemia inhibitory factor (LIF) (Murakami et al., 2014). These observations indicate that topographical microenvironments and chemokine gradients are established upon decidualization, which likely promote and direct trophoblast migration toward the spiral arterioles. Initially, invasion of the vessels results in plugging of these arteries by trophoblast. This facilitates conceptus development under low oxygen conditions, shielded from various environmental stressors. Notably, RNA-sequencing demonstrated that SUSD2 + stromal cells highly express genes encoding prototypic pericyte markers, including platelet-derived growth factor receptor (PDGFRB), CD146 (MCAM), neural/glial antigen 2 (CSPG4), and α smooth muscle actin (ACTA2) (Armulik et al., 2011; Ferland-McCollough et al., 2017). Hence, hereafter SUSD2+ cells are termed perivascular cells (PVC) whereas SUSD2− stromal cells are referred to as endometrial stromal cells (EnSC). On average 6 and 94% of non-immune stromal cells isolated from midluteal biopsies are PVC and EnSC, respectively (Lucas et al., 2016).

The metabolome encompasses a plethora of small organic molecules below 1.5 kDa with diverse physical and chemical structures, including nucleosides, amino acids, carbohydrates, and lipids (Cui et al., 2018). Exometabolomics, also known as metabolic footprinting, is the study of how cells transform their surrounding microenvironment through the secretion of metabolites. Exometabolomics have been used to study the secreted metabolome profile of micro-organisms, with mammalian exometabolomics being a nascent field (Birkenstock et al., 2012; Su et al., 2016). To date, exometabolomics has been performed to understand differences in profile of breast cancer cell lines, T-cell immunity, and stem cells (Ferreiro-Vera et al., 2011; Garcia-Manteiga et al., 2011; Matta et al., 2017). However, the exometabolome has not been established during decidualization. We hypothesize that specific temporally regulated secreted metabolites rapidly change upon decidualization, and that PVC and EnSC have distinct exometabolomes thereby establishing distinct microenvironmental niches.

In this study, we used both untargeted and targeted LC-MS exometabolomics to map the dynamic changes in the metabolic footprints of 12 paired PVC and EnSC cultures decidualized over an 8-day time-course.

## Materials and Methods

### Patient Selection and Endometrial Tissue Collection

The study was approved by the NHS National Research Ethics – Hammersmith and Queen Charlotte’s and Chelsea Research Ethics Committee (1997/5065). Endometrial biopsies from 12 patients were obtained in the Implantation Clinic, a specialized research clinic at University Hospitals Coventry and Warwickshire National Health Service Trust. Written informed consent was given by all participants in accordance with The Declaration of Helsinki 2000. Endometrial biopsies, timed 6 to 10 days following the preovulatory luteinizing hormone surge, were taken using a Wallach EndocellTM sampler (Wallach, Trumbull, United States) following a transvaginal ultrasound scan to exclude overt uterine pathology. Further, none of the participants had been prescribed hormonal treatment in at least 3 months prior to biopsy. Demographic details are summarized in Supplementary Table 1.

### Isolation of PVC and EnSC From Endometrial Biopsies

Single-cell suspensions of EnSC were isolated from 12 midluteal biopsies as described previously (Masuda et al., 2012). In short, samples were collected in DMEM/F-12 with 10% dextran coated charcoal activated fetal bovine serum (DCC-FBS), finely chopped, and enzymatically digested with deoxyribonuclease type I (0.1 mg/mL; Roche, Burgess Hill, United Kingdom) and collagenase (0.5 mg/mL; Sigma-Aldrich, Gillingham, United Kingdom) for 1 h at 37°C. The cells were passed through a 40 μm cell strainer (Fisher Scientific, Loughborough, United Kingdom), which retained epithelial cells more resistant to enzymatic digestion. Ficoll-Paque PLUS (GE Healthcare, Little Chalfont, United Kingdom) was utilized to remove erythrocytes from the stromal cell fraction. Subsequently, SUSD2+ and SUSD2**−** cells were isolated using magnetic bead sorting, as detailed previously (Masuda et al., 2012). Briefly, up to 1 × 10^6^ EnSC/100 μL of Magnetic Bead Buffer (0.5% BSA in PBS) were combined with 5 μL/1 × 10^6^ cells phycoerythrin (PE) conjugated anti-human SUSD2 antibody (BioLegend, London, United Kingdom). After 20 min on ice, approximately 1 × 10^7^ cells/80 μL of Magnetic Bead Buffer were incubated with anti-PE-magnetic-activated cell sorting MicroBeads (20 μL/1 × 10^7^ cells; Miltenyi Biotec, United Kingdom) for another 20 min. Cell suspensions (up to 1 × 10^8^ cells/500 μL of Magnetic Bead Buffer) were applied onto MS columns (Miltenyi Biotec) in a magnetic field, and washed multiple times with 500 μL of Magnetic Bead Buffer. SUSD2**−** cells passed freely through the column, whereas magnetically labeled SUSD2+ cells were retained. Following removal from the magnetic field, SUSD2+ cells were eluted from the columns with 1 mL of Magnetic Bead Buffer. Purified SUSD2**−** and SUSD2+ cells were expanded in DMEM/F12 with 10% DCC-FBS, 1% antibiotic-antimycotic solution (Invitrogen), 1% L-glutamine (Invitrogen), estradiol (1 nM; Sigma-Aldrich), insulin (2 μg/mL; Sigma-Aldrich), and basic fibroblast growth factor (10 ng/mL; Merck Millipore, Watford, United Kingdom). Decidualization experiments were carried out at passage 2. Briefly, paired SUSD2**−** and SUSD2 + cells were seeded 6-well plates at a density of 2 × 10^5^ cells/well and decidualized when confluent with 0.5 mM 8-bromoadenosine cAMP (8-bromo-cAMP; Sigma-Aldrich) and 1 μM medroxyprogesterone acetate (MPA; Sigma-Aldrich) in phenol red-free DMEM/F12 containing 2% DCC-FBS, 1% antibiotic-antimycotic solution. The spent medium was collected every 48 h for analysis, stored at −80°C and the differentiation medium refreshed. Decidual status was confirmed using transcript levels of the decidual marker gene *PRL*.

### Immunofluorescence

Immunostaining was completed at passage 1. Cells were fixed with 4% paraformaldehyde (Sigma-Aldrich) for 10 min and blocked with 3% BSA or 5% normal goat serum in PBS for 30 min. Cells were then incubated with an anti-human VAP-1 antibody (HPA000980, 1:100; Sigma Aldrich) for 1 h at room temperature, followed by incubation with Alexa Fluor 488 anti-rabbit secondary antibody for 1 h at RT (1:1000; Thermo Fisher Scientific). Slides were mounted with VECTASHIELD anti-fade mounting media with DAPI (Vector Laboratories) to visualize the nuclei. Images were captured using a Zeiss Laser Scanning Microscope LSM 510 at 60× magnification.

### Quantitative Reverse Transcription Polymerase Chain Reaction (RT-qPCR)

Total RNA was extracted from EnSC and PVC cultures using RNA STAT-60 (AMS Biotechnology). Equal amounts of total RNA (1 μg) were treated with DNase and reverse transcribed using the QuantiTect Reverse Transcription Kit (Qiagen), and the resulting cDNA was used as template in RT-qPCR analysis. Detection of gene expression was performed with Power SYBR Green Master Mix (Thermo Fisher Scientific), and the 7500 Real-Time PCR System (Applied Biosystems). The expression levels of the samples were calculated using the ΔΔCt method, incorporating the efficiencies of each primer pair. The variances of input cDNA were normalized against the levels of the L19 housekeeping gene. All measurements were performed in triplicate. Melting curve analysis confirmed amplification specificity. Primer sequences used were as follows: AOC3, forward: TCC TGT GCC AGG ACT CTCTT and reverse CAA GGT TCA GTG TCC CCT GT; L19, forward: GCG GAA GGG TAC AGC CAA T and reverse: GCA GCC GGC GCA AA; PRL, sense 5′-AAG CTG TAG AGA TTG AGG AGC AAA C-3′, PRL antisense 5′-TCA GGA TGA ACC TGG CTG ACT A-3′.

### Preparation of Spent Medium for Analysis

For untargeted exometabolome analysis, samples were prepared as described previously (Zhu et al., 2011; Cui et al., 2013) with some modifications. Briefly 50 μL of EnSC and PVC conditioned media were thawed at 4°C, and quality control (QC) samples were prepared by mixing an equal amount of conditioned media from each of the samples. Both QC and the conditioned media from PVC and EnSC were processed in the same way. Proteins were precipitated with 200 μL ice-cold methanol containing 10 μg/mL 9-fluorenylmethoxycarbonyl-glycine as an internal standard (ISTD), and vortexed. Following centrifugation at 16,000 rpm for 10 min at 4°C, the supernatant was collected and evaporated until dry in a speed vacuum evaporator then resuspended in 200 μL of water/methanol (98:2; v/v) for liquid chromatography-mass spectrometry (LC-MS) analysis. All samples were kept at 4°C and analyzed within 48 h. The sample run order was randomized to remove batch effects and the QC samples were analyzed after every eight samples to monitor the stability of the system.

### Untargeted LC-MS Exometabolomics

Untargeted exometabolomics was performed as previously described (Cui et al., 2017). The prepared conditioned media was analyzed using Agilent 1290 ultrahigh-pressure liquid chromatography system (Waldbronn, Germany) equipped with a 6520 QTOF mass detector managed by a MassHunter workstation. The oven temperature was set at 45°C.

The 5 μL of injected sample was separated using an Agilent rapid resolution HT Zorbax SB-C18 column (2.1 × 50 mm, 1.8 mm; Agilent Technologies, Santa Clara, CA, United States), 0.4 mL/min flow rate, and a gradient elution involving a mobile phase consisting of (A) 0.1% formic acid in water and (B) 0.1% formic acid in methanol. To start the mobile phase was set at 5% B, with a 7-min linear gradient to 70% B, then a 12 min gradient to 100% B. This was held for 3 min then returned to 5% B in 0.1 min.

Both positive and negative electrospray ionization were used to collect mass data between m/z 100 and 1000 at a rate of two scans per second. The ion spray was set at 4,000 V, and the heated capillary temperature was maintained at 350°C. The drying gas and nebulizer nitrogen gas flow rates were 12.0 L/min and 50 psi, respectively. Two reference masses (m/z 121.0509 (C5H4N4) and m/z 922.0098 (C18H18O6N3P3F24) were continuously infused to the system to allow constant mass correction during the run.

### Targeted LC-MS Exometabolomics

Adenosine, inosine, hypoxanthine, and xanthine were measured and analyzed by targeted LC-MS/MS analysis. The analysis was conducted as described previously with some modifications (Garcia-Manteiga et al., 2011). Briefly, LC-MS analysis was performed with Agilent 1290 ultrahigh-pressure liquid chromatography system (Waldbronn, Germany) coupled to an electrospray ionization with iFunnel Technology on an Agilent 6490 triple quadrupole mass spectrometer.

The auto-sampler was cooled at 4°C and 2 μL of injected sample was chromatographically separated using an Atlantis HILIC column (2.1 × 100 mm; Waters, Eschborn, Germany). The mobile phases were (A) 10 mM ammonium formate and 0.1% formic acid in water and (B) 0.1% formic acid in acetonitrile. Initially, 100% was utilized for 2 min, then reduced to 80% in a linear gradient for 11 min, and to 40% B over 1 min. This was held for 5 min and then the mobile phase was returned to starting conditions over 6 min. The column was kept at 45°C and the flow rate was 0.4 mL/min. Direct infusion of individual standard solutions allowed optimization of both the mass transition and collision energy for each compound by direct infusion. Both positive and negative electrospray ionization modes were performed with the following source parameters: drying gas temperature at 250°C with a flow of 14 L/min, sheath gas temperature at 400°C with a flow of 11 L/min, nebulizer gas pressure at 40 psi, capillary voltage 4,000 V and 3,500 V for positive and negative mode, respectively, and nozzle voltage 500 V for both positive and negative modes.

### Data Analysis

Raw spectrometric data in untargeted metabolomics were analyzed by MassHunter Qualitative Analysis software (Agilent Technologies, United States). The Molecular Feature Extractor algorithm was used to obtain the molecular features, employing the retention time (RT), chromatographic peak intensity and accurate mass as input. Next, the features were analyzed by MassHunter Mass Profiler Professional software (Agilent Technologies, United States). Here, only features detected in at least 80% of the samples at the same sampling time point signal with an intensity ≥20,000 counts, three-times the limit of detection of the LC-MS instrument, were kept for further processing. Pre-statistical filtering of samples was performed, including the removal of samples with more than 60% missing data. One sample had 62% missing data and was subsequently removed from the untargeted analysis. The tolerance window for alignment of RT and m/z values was set at 0.15 min and 2 mDa, respectively, and the data normalized to the 9-fluorenylmethoxycarbonyl-glycine ISTD spike.

Raw spectrometric data in targeted metabolomics were processed using MassHunter Workstation Quantitative Analysis software (Agilent Technologies, United States). Missing values were imputed using half the lowest value. For both untargeted and targeted analysis, metabolites that were differentially expressed were inputted into the Metaboanalyst Pathway Analysis Tool to identify pathways that were altered during decidualization or between PVC and EnSC. This incorporates both pathway topology and enrichment analysis to output pathways that have changed significantly, and the pathway impact (Chong et al., 2019). The hypergeometric test was used and relative-betweenness centrality for over-representation analysis and pathway topology analysis, respectively. The pathway impact is calculated by the number of compounds that are significantly altered in relation to the total number of compounds in the pathway (Chong et al., 2019).

### Statistical Analysis

For statistical analysis, non-parametric Test (Wilcoxon, Mann–Whitney test) with Holm-Sidak Multiple Testing Correction was employed and statistical significance was set at *p* < 0.05. For multivariate data analysis using hierarchical clustering or partial least squares regression (PLSR) analysis, data were normalized by applying log2, median-centering and dividing by standard deviation. Unsupervised Euclidean distance hierarchical clustering was performed using HemI (Deng et al., 2014). In addition, fold change (FC) analysis was performed to further filter the features and only those features with FC > 1.5 were selected as potential significantly altered metabolites across decidualization.

### Compound Identification

The identification of the differential metabolites structure was based on our published work (Cui et al., 2017). Briefly, MassHunter software (Agilent) was used to calculate the elemental compositions of the metabolites based on their exact mass, the isotope pattern, and the nitrogen rule. The elemental composition in combination with the exact mass were used in open-source database searches, including HMDB^1^, LIPIDMAPS^2^, MassBank^3^, and METLIN^4^. Next, MS/MS experiments were performed to obtain structural information from the fragmentation pattern of the metabolite and these MS/MS spectra were searched and compared to compounds in the databases. Finally, the metabolites were confirmed by comparison with the standards where commercially available. The metabolites meet the minimum reporting standards for chemical analysis in metabolomics recommended by Metabolomics Standard Initiative (MSI) (Sumner et al., 2007).

## Results

### Exometabolic Footprints of Decidualizing Primary EnSC

**FIGURE 1:**
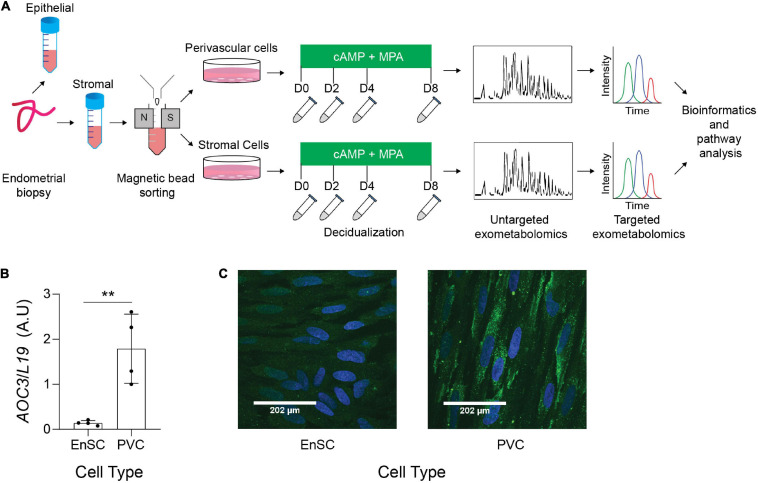
Exometabolic footprints of decidualizing primary EnSC **(A)** Schematic representation of experimental design and the analytical process. Paired PVC and EnSC cultured were established from 12 endometrial biopsies. Conditioned media was collected from undifferentiated cultures (D0, day 0) and after 2, 4, and 8 days of decidualization. **(B)** Quantitative real-time PCR measuring *AOC3* mRNA in PVC and EnSC. **(C)** Immunofluorescence staining of VAP-1 (green) in PVC and EnSC, following magnetic-activated sorting. DAPI (blue) was utilized to stain the nuclei. Pictures were taken at 60× magnification with a Zeiss Laser Scanning Microscope LSM 510. Scale bar represents 160 pixels. **p < 0.01.

The endometrial stromal fraction of 12 midluteal biopsies were separated into SUSD2+ PVC and SUSD2**−** EnSC by magnetic-activated cell sorting (MACS) (Figure 1). These cells were tested for AOC3/VAP-1 expression, as we have previously reported that AOC3 is highly enriched in PVC cells (Murakami et al., 2014). AOC3 mRNA levels were significantly enriched in freshly isolated PVC cells when compared to their EnSC counterparts (*p* = 0.0052; Figure 1B). Immunofluorescence showed VAP-1 immunoreactivity on the cell surface of SUSD2 + cells whereas more discrete, punctate cytoplasmic staining was observed in EnSC (Figure 1C and Supplementary Figures 1, 2). Both subpopulations were cultured to confluency and then decidualized over 8 days. Conditioned culture media of paired decidualizing PVC and EnSC cultures were collected every 48 h and subjected LC-MS based metabolomics.

A total of 145 annotated secreted metabolites and 5 unidentified metabolites were detected in the conditioned media of both PVC and EnSC (Supplementary Table 2). There were no metabolites unique to either PVC or EnSC. Hence, the metabolite data of PVC and EnSC were first combined to construct a temporal map of metabolic footprints associated with decidualization.

**FIGURE 2:**
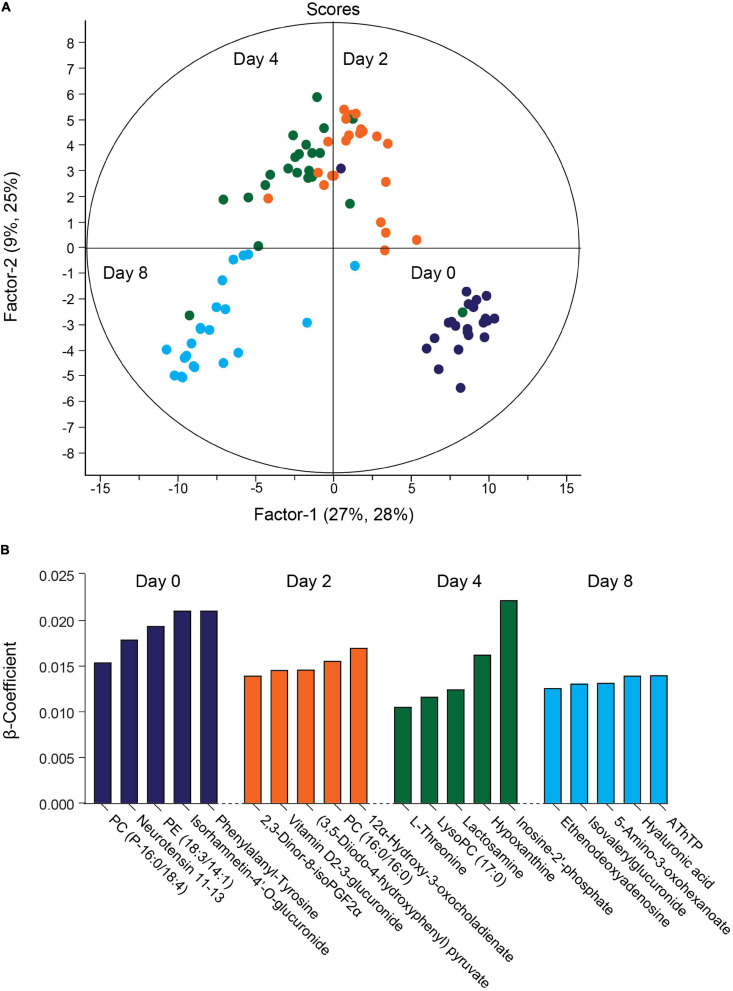
Temporal exometabolome of EnSC. **(A)** Partial least squares regression (PLSR) analysis separated the exometabolomes according to decidual time-points. The secreted metabolite levels were log2 transformed, the data centered using median, and scaled by SD. **(B)** Metabolites with the top five PLSR regression coefficients across the decidual time-course.

The QC samples clustered together in principal component analysis (PCA) scores plots (Supplementary Figure 3), indicating good stability and reproducibility of the chromatographic separation throughout the entire sequence. PLSR analysis separated the decidual time points into distinct groups (Figure 2A). Regression coefficients were used to identify metabolites that shaped the progression of decidualizing cells throughout the time-course. Metabolites with the five highest β-coefficient (β) at each timepoint are depicted in Figure 2B. Phenylalanyl-tyrosine (β: 0.02), 2,3-dinor-8-iso prostaglandin F2α (2,3-dinor-8-isoPGF2α) (β: 0.01), and hypoxanthine (β: 0.02) were identified, respectively, as influencers on day 0 (undifferentiated cells), day 2 and day 4 of the decidual time-course. Adenosine thiamine triphosphate (AThTP) (β: 0.01) and hyaluronic acid (β: 0.01) were the highest influencers of day 8 of decidualization.

**FIGURE 3:**
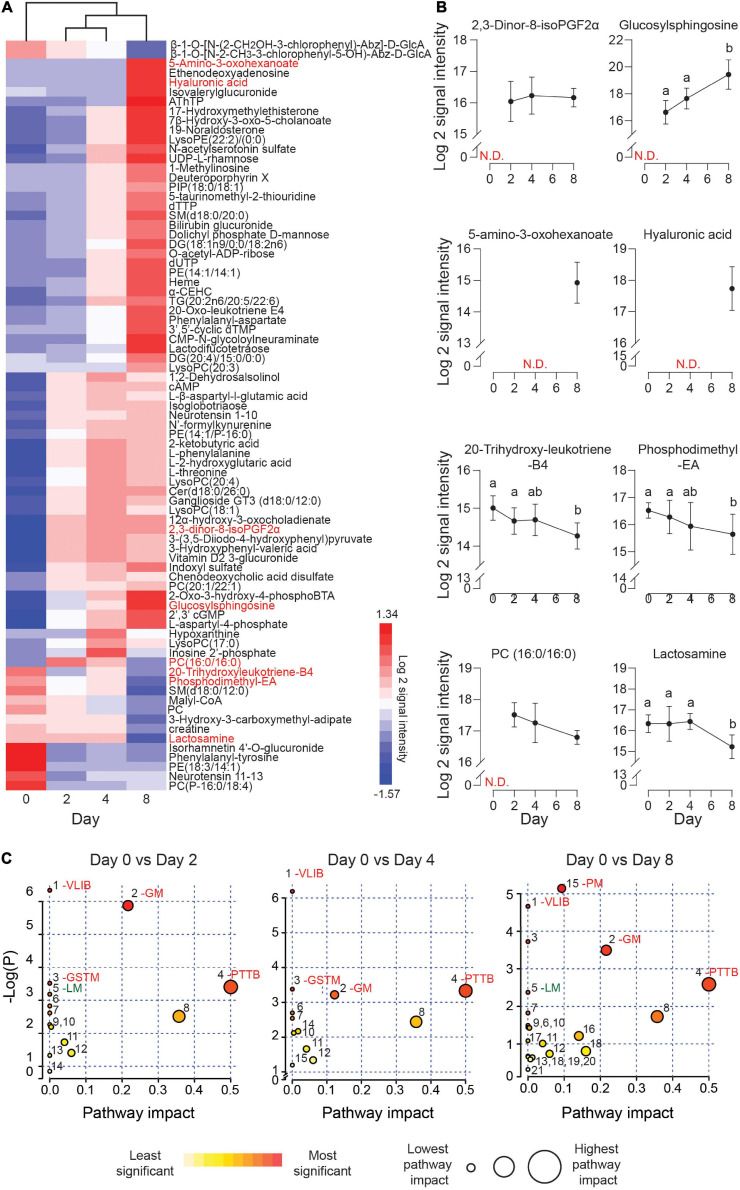
Exometabolic footprints of decidualizing primary EnSC. **(A)** Heatmap of differentially secreted metabolites across the time-course based on unsupervised Euclidean distance hierarchical clustering. **(B)** Representative metabolites altered upon decidualization. Data represent mean ± SD. N.D, not detected. Different letters above the SD indicates differential secretion at the indicated time-point (*p* < 0.05; *t*-test with Holm-Sidak correction). **(C)** MetaboAnalyst pathway analysis across decidualization in EnSC. The most enriched pathways include VLIB, valine, leucine, and isoleucine biosynthesis; GM, glycero-phospholipid metabolism; GSTM, glycine, serine, and threonine metabolism; PTTB, phenylalanine tyrosine and tryptophan biosynthesis; LM, linoleic acid metabolism; and PM, pyrimidine metabolism. Other pathways are numbered and tabulated in Supplementary Figure 5.

The exometabolome analysis of conditioned media revealed a marked temporal change in metabolic footprints upon decidualization. Out of the 150 detected compounds, the secreted levels of 79 metabolites changed by 1.5-fold (*p* < 0.05) over the 8-day decidual time-course (Figure 3A and Supplementary Table 3). Unsupervised hierarchical clustering showed a major bifurcation in exometabolome profiles between undifferentiated and decidualizing cells. Further, each decidual time-point was characterized by a unique footprint, which supports the notion that decidualization is a multistep differentiation process (Figure 3A).

Figure 3B highlights several regulated secreted metabolites, some of which are already implicated in decidualization. For example, the prostaglandin metabolite, 2, 3-dinor-8-isoPGF2α, which can be derived from PGE2, was undetectable in undifferentiated cultures but consistently upregulated in differentiating cells throughout the decidual time-course. Secreted levels of glucosylsphingosine, a sphingolipid metabolite, followed the same pattern, in keeping with the dependency of the decidual process on *de novo* sphingolipid synthesis (Mizugishi et al., 2007; Ding et al., 2018). Several metabolites were selectively secreted by the decidualizing cells on day 8, including 5-amino-3-oxohexanoate and hyaluronic acid (HA). HA prevents apoptosis of decidual cells through binding to its receptor CD44 (Takizawa et al., 2014). 20-trihydroxy-leukotriene-B4 and PE (18:3/14:1) are examples of metabolites whose extracellular concentrations declined upon decidualization, whereas phosphatidylcholine (PC) (16:0/16:0) and lactosamine exemplify metabolites exhibiting a biphasic secreted pattern, peaking on day 2 and day 4 of decidualization, respectively. A difference in molecular mass distinguishes endogenous cyclic AMP levels which increased upon decidualization from the 8-bromo-cAMP utilized to stimulate decidualization.

Metaboanalyst was used to identify metabolic pathways regulated upon decidualization (Chong et al., 2019). Twenty-one metabolic pathways were identified across the decidual time-course (Supplementary Figure 4). As shown in Figure 3C, prominent pathways enriched across the decidual time-course included valine, leucine, and isoleucine biosynthesis (VLIB), glycerophospholipid metabolism (GM), and phenylalanine, tyrosine, and tryptophan metabolism (PTTM). Pyrimidine metabolism (PM) was enriched on day 8 of decidualization, suggesting the requirement for pyrimidines as nitrogenous bases in DNA and RNA.

### Metabolites Differentially Secreted by Decidualizing PVC and EnSC

**FIGURE 4:**
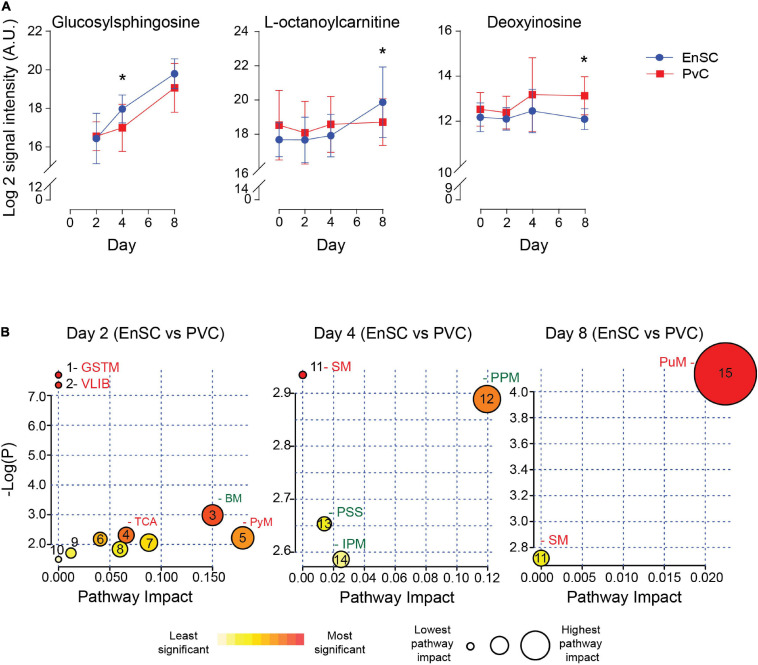
Metabolites differentially secreted by decidualizing PVC and EnSC. **(A)** Compounds significantly different between PVC and EnSC. Data represents mean ± SD. * indicates *p* < 0.05 (*t*-test with Holm-Sidak correction). **(B)** Metaboanalyst Pathway analysis comparing PVC to EnSC of untargeted metabolomics by for day 2, 4, and 8 decidualization. Pathways labeled: GSTM, glycine, serine, and threonine metabolism; VLIB, valine, leucine, and isoleucine biosynthesis; BM, biotin metabolism; TCA, tricarboxylic acid cycle; PyM, pyruvate metabolism; SM, sphingolipid metabolism; PPM, pentose phosphate metabolism; PPS, phosphatidylinositol signaling system; IPM, inositol phosphate metabolism; and PuM, purine metabolism. Other pathways, represented by numbers, can be found in Supplementary Figure 6.

The temporal changes in the metabolic footprints of decidualizing PVC and EnSC were comparable but not identical, as demonstrated PLSR analysis (Supplementary Figure 5). Notably, apart from a single metabolite (N2, N2-Dimethylguanosine), no significant difference was observed in the exometabolomes of undifferentiated PVC and EnSC. By day 2 of decidualization, however, the secreted levels of 12 annotated metabolites were significantly higher in PVC compared to EnSC and 4 were lower (1.5 fold-change, *p* < 0.05; Supplementary Table 4). By day 4 and day 8 of decidualization, secreted levels of 6 and 9 metabolites, respectively, were significantly different between PVC and EnSC (Supplementary Table 4). Out of a total of 32 differentially secreted, annotated metabolites, 20 were more abundantly expressed by PVC. Figure 4A shows examples of metabolites differentially secreted between PVC and EnSC at each timepoint of the decidual pathway.

To further elucidate the metabolic differences between PVC and EnSC upon decidualization, pathway enrichment analysis was performed (Figure 4B). Interestingly, distinct metabolomic profiles emerged. The tricarboxylic acid cycle (TCA), and glycine, serine and threonine metabolism (GSTM) were enriched in PVC cells on day 2 of decidualization. Enrichment of sphingolipid metabolism (SM) arose at in PVC at day 4, whereas inositol phosphate metabolism (IPM) and the pentose phosphate metabolism (PPM) were more prominent in EnSC. Finally, on day 8, sphingolipid (SM) and purine metabolism (PuM) were enriched in PVC. Purine metabolism had the highest pathway impact and significance (Figure 4B and Supplementary Figure 6), suggesting a greater autocrine or paracrine requirement of purine signaling upon decidualization in PVC when compared to EnSC.

### Targeted Exometabolomic Analysis Validates Altered Purine Metabolite Secretion Upon Decidualization

**FIGURE 5:**
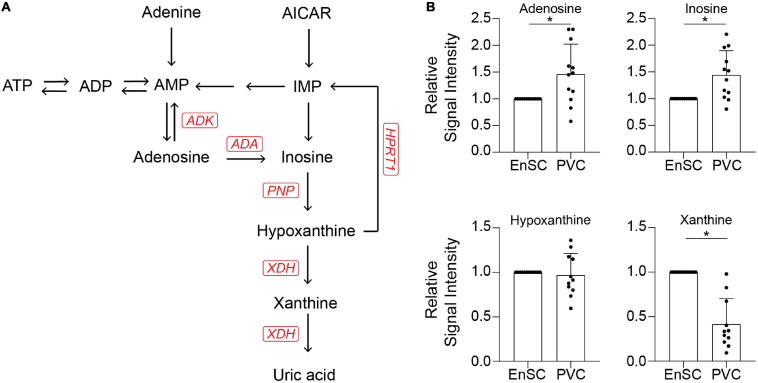
Targeted exometabolomic analysis validates altered purine metabolite secretion at day 8 of decidualization. **(A)** Schematic overview of the purine metabolic pathway. **(B)** Secreted purine metabolites were measured by targeted mass spectrometry in conditioned medium of paired PVC and EnSC cultures decidualized for 8 days. The data show the relative change in secreted metabolite levels in PVC compared to EnSC cultures. The data are mean ± SD and the individual data points show variability between paired cultures. * indicates *p* < 0.05 (*t*-test with Holm-Sidak correction).

The enrichment in purine metabolism (Figure 5A) prompted us to investigate its wider metabolomic network in greater depth. We developed an analytically robust targeted mass spectrometry analysis (average coefficient of variation = 23.9%) to assess adenosine, inosine, hypoxanthine, and xanthine levels. Adenosine (7.8 fold-change, *p* = 0.0001), inosine (1.53 fold-change, *p* < 0.02) and xanthine (3.1 fold-change, *p* = 0.0001) were significantly higher in PVC on day 8 of the decidual time-course in comparison to undifferentiated cells whereas uridine (0.21 fold-change, *p* < 0.0001) and cytidine (0.42 fold-change, *p* = 0.001) were significantly lower. Furthermore, xanthine levels were significantly lower in PVC compared to EnSC conditioned media in both undifferentiated and decidualized cultures (Figure 5B and Supplementary Table 5).

## Discussion

Several studies have documented the dynamic changes in the EnSC transcriptome, proteome and secretome upon decidualization (Garrido-Gomez et al., 2011; Murakami et al., 2014; Rytkönen et al., 2019), but changes in secreted metabolites that may function as signaling molecules have not yet been characterized. Understanding the niche signaling enhances our understanding of human implantation and pregnancy. Here, we demonstrate that decidualization of EnSC and PVC induces conspicuous metabolic footprints that are tightly regulated in a temporal fashion. For instance, HA secretion was prominent upon full decidualization (day 8) in both EnSC and PVC. This ECM macromolecule has gel properties and is typically highly expressed in rapidly expanding tissues (Karousou et al., 2017). The temporally restricted pattern of HA secretion upon decidualization, therefore, suggests a potential role in decidual expansion in early pregnancy. HA also binds CD44 expressed on extravillous trophoblast and vasculature, indicating a role in spiral artery remodeling (Takizawa et al., 2014). Moreover, loss of HA secretion has been linked to miscarriage (Takizawa et al., 2014), which further underscores the importance of appropriate temporospatial secretion of this ECM component in pregnancy.

Our untargeted analysis also revealed significant changes in secreted lipid metabolites upon decidualization. Phosphodimethyl-EA and PC (16:0/16:0) are present in phospholipid bilayers, act as signaling molecules, and contribute phosphoethanolamine and phosphocholine, respectively, to form sphingolipids such as sphingomyelin (Gault et al., 2010; Merrill, 2011; Furse and de Kroon, 2015). Decreasing levels of phosphodimethyl-EA, PC (16:0/16:0) upon decidualization occurred in concert with increased secretion of glucosylsphingosine, indicating a role for extracellular sphingolipids. Enhanced sphingolipid secretion corroborates recent findings from mice demonstrating that genes encoding sphingolipid producing enzymes are upregulated upon decidualization (Ding et al., 2018). Mice deficient in sphingolipid synthesizing enzymes exhibit impaired decidualization, reduced implantation sites, and vascular endothelial defects that compromise trophoblast from invading the maternal vasculature (Mizugishi et al., 2007; Ding et al., 2018). Sphingolipids play a role in angiogenesis and promote phospholipase A2 enzymatic activity, the enzyme required to form arachidonic acid, a precursor for prostaglandins and leukotrienes (Murakami and Kudo, 2002; Singh and Subbaiah, 2007). While 20-trihydroxyleukotriene secretion decreased upon decidualization, 2,3-dinor-8-isoPGF2α levels were consistently elevated across the time-course, suggesting that sustained *de novo* synthesis of sphingolipids is important for decidualization, as demonstrated in mice (Ding et al., 2018). Alterations in pyrimidine and glycerolipid metabolism coincides with morphological changes observed during decidualization. Enrichment of glycolipids metabolism across decidualization, a precursor to fatty acid production, is consistent with the accumulation of lipid droplets, a key energy store, upon decidualization (Guo et al., 2009; Kuroda et al., 2013; Gellersen and Brosens, 2014), whereas pyrimidines are required for nucleic acid production and as an energy carrier in differentiation (Lane and Fan, 2015).

Exometabolomic differences were also observed between PVC and EnSC. The most notable differences occurred on day 2 of decidualization, i.e., coinciding with the initial inflammatory phase (Salker et al., 2012; Stadtmauer and Wagner, 2020). The secreted response at this time-point was also more pronounced in PVC than EnSC, in keeping with the topology of decidualization *in vivo* (Murakami et al., 2014). The PVC exometabolome on day 2 was enriched in higher energy metabolites linked to pyruvate metabolism and TCA cycle, indicative of Warburg effect or active metabolism (DeBerardinis and Chandel, 2020). On day 8 of the decidual time-course, the PVC exometabolome was enriched for other metabolites, such as sphingolipids and purines involved in signaling and structural integrity (Merrill, 2011; Linden et al., 2019).

Purines, along with pyrimidines, stimulate purinergic receptors, which belong to two subfamilies, the P2X and P2Y receptors (P2XR and P2YR, respectively). P2XR are plasma membrane channels that increase cytosolic Ca^2+^ concentration and mediate the flux of K^+^ and Na^+^ upon activation (di Virgilio and Adinolfi, 2017). P2 × 4R is linked to PGE2 signaling (Ulmann et al., 2010). P2YR are G-protein coupled receptors that modulate Ca^2+^ mobilization and cAMP signaling in response to either the pyrimidines adenine and uridine, or purines. The affinity for different ligands varies between receptors; for example, P2Y2R preferentially binds UTP, whereas P2Y11R binds ATP (Giuliani et al., 2019).

Purines modulate cell growth, act as coenzymes, and are a source of energy whereas pyrimidines contribute to phospholipid biosynthesis, glycosylation and detoxification (Löffler et al., 2005; Yin et al., 2018). Not only do endometrial cells, and particularly epithelial cells, express purinergic receptors but placental trophoblast expresses nearly the entire repertoire of purinergic receptors (Roberts et al., 2006, 2007; Burnstock, 2014). Thus, purine and pyrimidine nucleotides potentially act as metabolic signals acting on the purinergic receptors at the placental-decidual interface. In concurrence, purine derivatives are over-represented in bovine conceptus elongation, further emphasizing the importance of purines in conceptus development (Simintiras et al., 2019).

Intriguingly, purine levels correlate with the induction of stress ligands expression, such as the cell surface glycoprotein MHC class I polypeptide-related sequence A (MICA), a ligand for the natural killer group 2D receptor (NKG2D) (McCarthy et al., 2018). Recently, uNK cells were shown to target and eliminate senescent decidual cells through activation of NKG2D receptors (Brighton et al., 2017; Lucas et al., 2020), a process that is purportedly essential to prevent chronic senescence of the placental-decidual interface in pregnancy, leading to tissue breakdown and miscarriage (Lucas et al., 2020).

Targeted LC-MS demonstrated markedly lower levels of xanthine in PVC compared to EnSC, irrespective of decidualization. By contrast, decidualized PVC secreted higher levels of adenosine and inosine. Adenosine is an anti-inflammatory metabolite and stress signal (Kaster et al., 2013), and precursor to inosine. Inosine, an immunomodulator, is exported from cells through nucleoside transporters when intracellular concentrations are high (Kaster et al., 2013). Extracellular inosine signals through adenosine receptors and is broken down to form the downstream metabolite hypoxanthine. Xanthine is formed during the breakdown of hypoxanthine by xanthine dehydrogenase (XDH). In keeping with our observations, XDH transcript levels are significantly lower in PVC compared to EnSC (Murakami et al., 2014). Lower xanthine secretion by PVC suggests that higher energy purines are important in the perivascular niche as hypoxanthine is readily available to form inosine, a paracrine signaling molecule that can be recycled back to produce upstream metabolites, such as ATP. Extracellular release of ATP can induce a pro-inflammatory state, regulate the binding activity of estrogen receptors, increase production of reactive oxygen species, and induce metalloproteinase expression (Chang et al., 2007; Cruz et al., 2007; Cekic and Linden, 2016). Furthermore, ATP has been shown to promote IL-8 secretion in endometrial epithelial cells and stimulate decidualization (Gu et al., 2020). Thus, redirecting hypoxanthine toward the upstream purines may represent a mechanism to heighten decidualization of PVC cells. Taken together, the data suggest that a balance and switch between ATP, a mainly proinflammatory molecule, and adenosine, an anti-inflammatory factor, is required for an effective decidual response. Our results reflect the exometabolomic footprint of decidualizing cells from women with fertility issues. Evaluation of other samples, including women with normal reproductive histories, are required to test generalizability. In summary, this study is the first to map the temporally resolved exometabolome changes in differentiating human EnSC and to highlight the potential of secreted metabolites acting as autocrine or paracrine signaling molecules as the decidual process unfolds. Although the overall temporal change in metabolic footprints was remarkably consistent between decidualizing PVC and EnSC, differential secretion of specific metabolites not only reflects metabolic differences between stromal subpopulations but also raises the possibility of spatial organization of metabolic cues at the decidual-placental interface.

## Data Availability Statement

The original contributions presented in the study are included in the article/Supplementary Material, further inquiries can be directed to the corresponding author/s.

## Ethics Statement

The studies involving human participants were reviewed and approved by the NHS National Research Ethics – Hammersmith and Queen Charlotte’s and Chelsea Research Ethics Committee (1997/5065). The patients/participants provided their written informed consent to participate in this study.

## Author Contributions

YL: conceptualization. JZ, SG, MD, EL, LC, and KM: research. YL, JB, and QC: resources and supervision. SH: data analysis. SH, YL, and JB: writing. All authors contributed to the article and approved the submitted version.

## Conflict of Interest

The authors declare that the research was conducted in the absence of any commercial or financial relationships that could be construed as a potential conflict of interest.

## Footnotes

1 http://www.hmdb.ca/

2 http://www.lipidmaps.org/

3 http://www.massbank.jp/

4 http://metlin.scripps.edu/
