# Supplementary material for: Exometabolomic Analysis of Decidualizing Human Endometrial Stromal and Perivascular Cells
Source: Front Cell Dev Biol. 2021 Jan 28;9:626619. doi: 10.3389/fcell.2021.626619 (PMC7876294; doi:10.3389/fcell.2021.626619)
Supplement: Supplementary file 1 [file Data_Sheet_1.PDF]

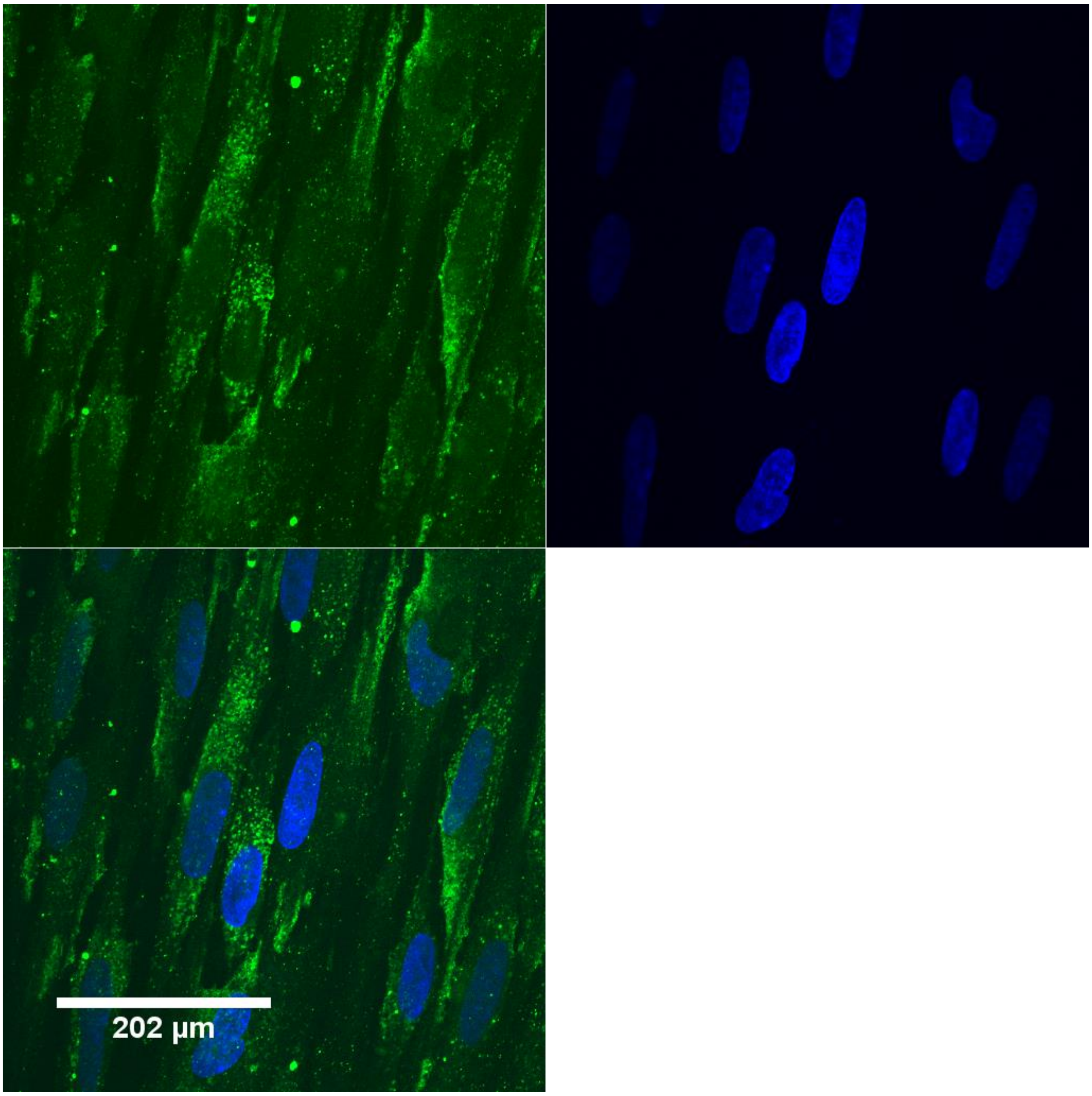

Figure S1: Confocal microscopy: perivascular marker VAP-1 (green) on cell surface of SUSD2+ cells. Image taken at 60 x magnification using a Zeiss Laser Scanning Microscope LSM 510. Nuclei visualized with DAPI (blue). Scale bar represents 202µm.
