# Supplementary material for: Exometabolomic Analysis of Decidualizing Human Endometrial Stromal and Perivascular Cells
Source: Front Cell Dev Biol. 2021 Jan 28;9:626619. doi: 10.3389/fcell.2021.626619 (PMC7876294; doi:10.3389/fcell.2021.626619)
Supplement: Supplementary file 2 [file Data_Sheet_2.PDF]

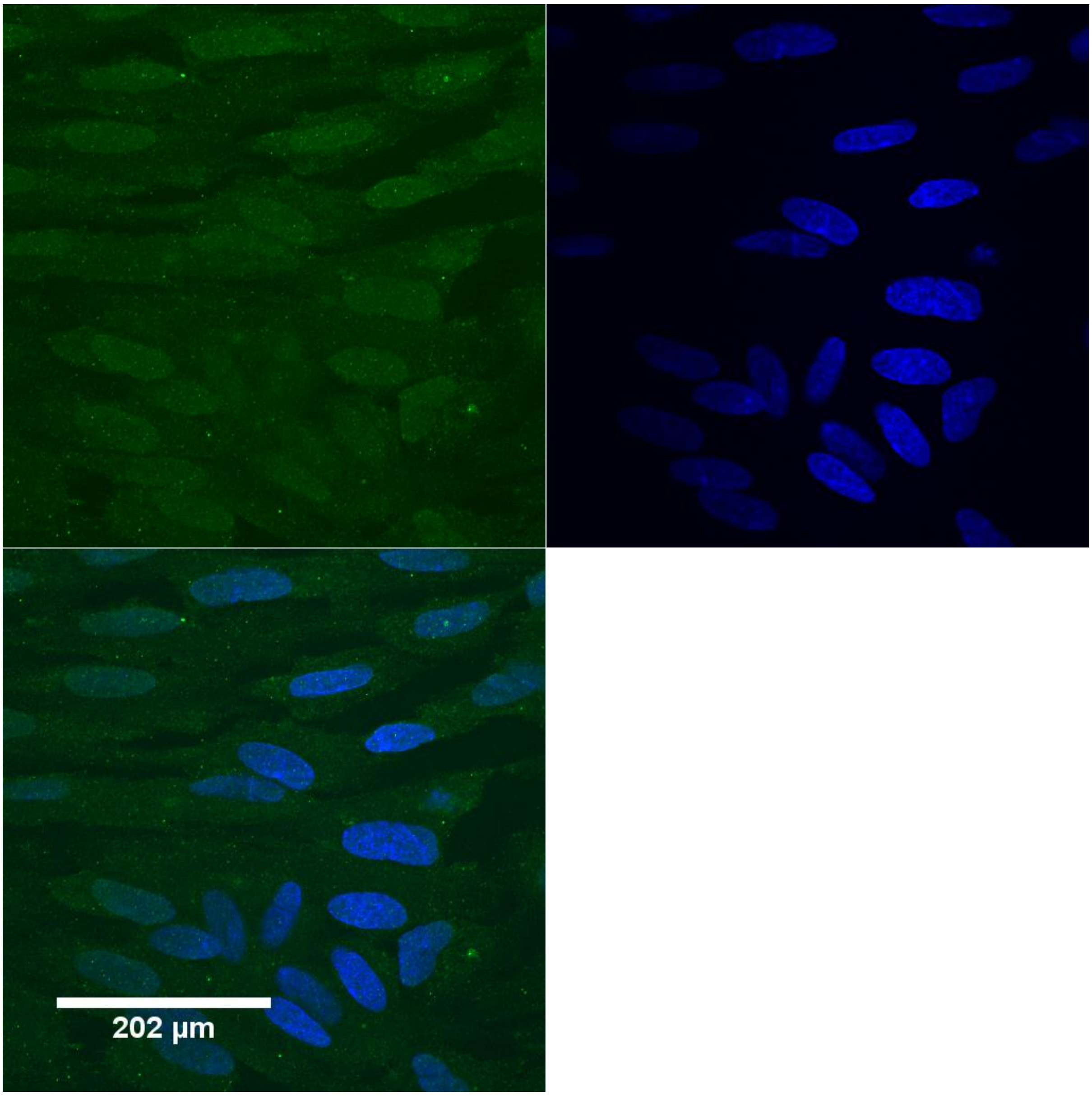

Figure S2: Confocal microscopy: perivascular maker VAP-1 (green); nuclei visualized with DAPI (blue). Image taken at 60 x magnification using a Zeiss Laser Scanning Microscope LSM 510. Scale bar represents 202µm.
