# Supplementary figures and images for: Exometabolomic Analysis of Decidualizing Human Endometrial Stromal and Perivascular Cells
Source: Front Cell Dev Biol. 2021 Jan 28;9:626619. doi: 10.3389/fcell.2021.626619 (PMC7876294; doi:10.3389/fcell.2021.626619)

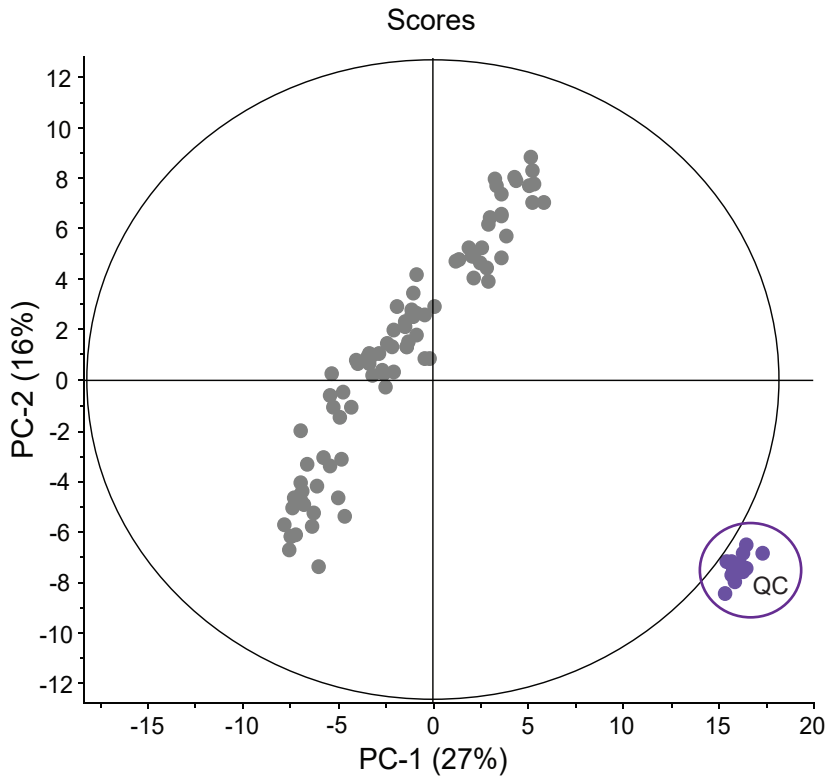

**Figure S3** Principal component analysis clusters quality control (QC) samples

Supplement: Supplementary file 3 [file Data_Sheet_3.PDF]
