# Supplementary material for: Exometabolomic Analysis of Decidualizing Human Endometrial Stromal and Perivascular Cells
Source: Front Cell Dev Biol. 2021 Jan 28;9:626619. doi: 10.3389/fcell.2021.626619 (PMC7876294; doi:10.3389/fcell.2021.626619)
Supplement: Supplementary file 4 [file Data_Sheet_4.PDF]

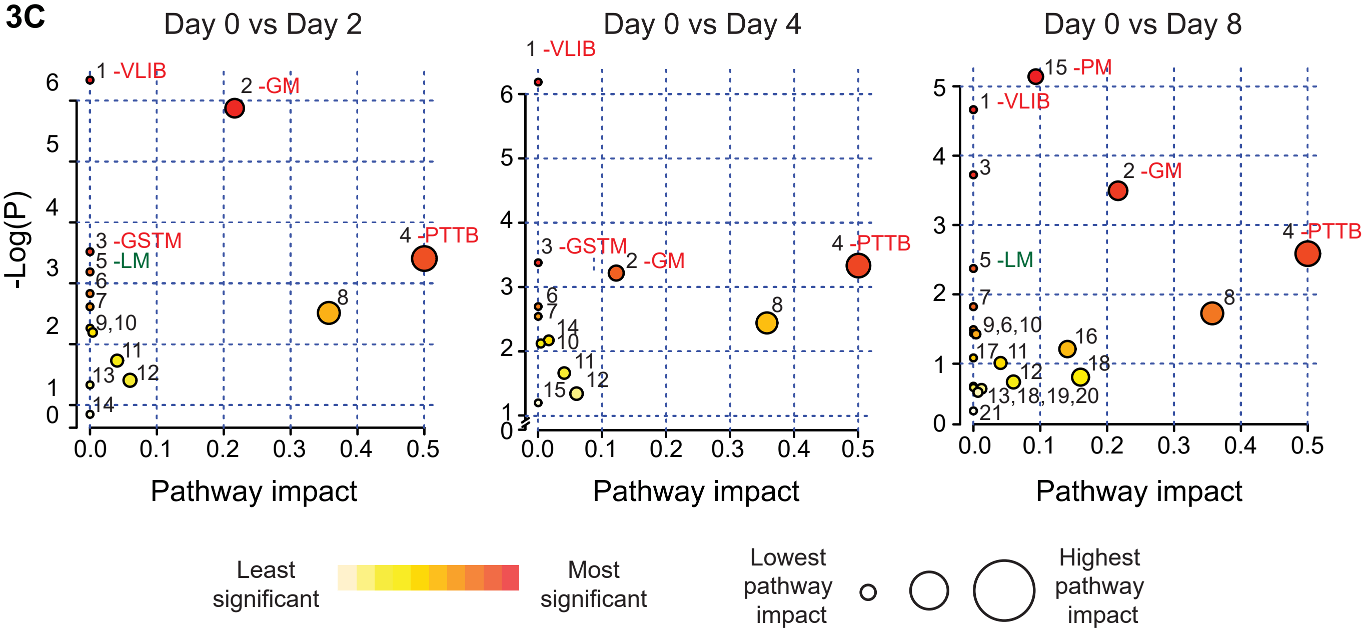

Number	Pathway
1	Valine, leucine & isoleucine biosynthesis
2	Glycerophospholipid metabolism
3	Glycine, serine & threonine metabolism
4	Phenylalanine, tyrosine, and tryptophan biosynthesis
5	Linoleic acid metabolism
6	Aminoacyl-tRNA biosynthesis
7	Vitamin B6 metabolism
8	Phenylalanine metabolism
9	alpha-Linolenic acid metabolism
10	Glycosylphosphatidylinositol (GPI)- anchor biosynthesis
11	Propanoate metabolism
12	Cysteine& methionine metabolism
13	Arachidonic acid metabolism
14	Purine metabolism
15	Pyrimidine metabolism
16	Pentose & glucuronate interconversions
17	Sphingolipid metabolism
18	Porphyrin & chlorophyll metabolism
19	Amino & nucleotide sugar metabolism
20	Arginine & proline metabolism
21	1 N-Glycan biosynthesis

Key
Higher levels at day zero
Lower levels at day zero

Note: Numbers correspond to numbers on Figure 3C, pathways that are altered upon decidualization i.e. Day 0 to Day 2, Day 0 to Day 4 or Day 0 to Day 8 decidualization

Figure S4: MetaboAnalyst pathway analysis across decidualization in EnSC. The most enriched pathways include valine, leucine, and isoleucine biosynthesis (VLIB); glycero-phospholipid metabolism (GM); glycine, serine, and threonine metabolism (GSTM); Phenylalanine tyrosine and tryptophan biosynthesis (PTTB); Linoleic acid metabolism (LM); and pyrimidine metabolism (PM). Other pathways are numbered and tabulated.
