# Supplementary material for: Exometabolomic Analysis of Decidualizing Human Endometrial Stromal and Perivascular Cells
Source: Front Cell Dev Biol. 2021 Jan 28;9:626619. doi: 10.3389/fcell.2021.626619 (PMC7876294; doi:10.3389/fcell.2021.626619)
Supplement: Supplementary file 5 [file Data_Sheet_5.PDF]

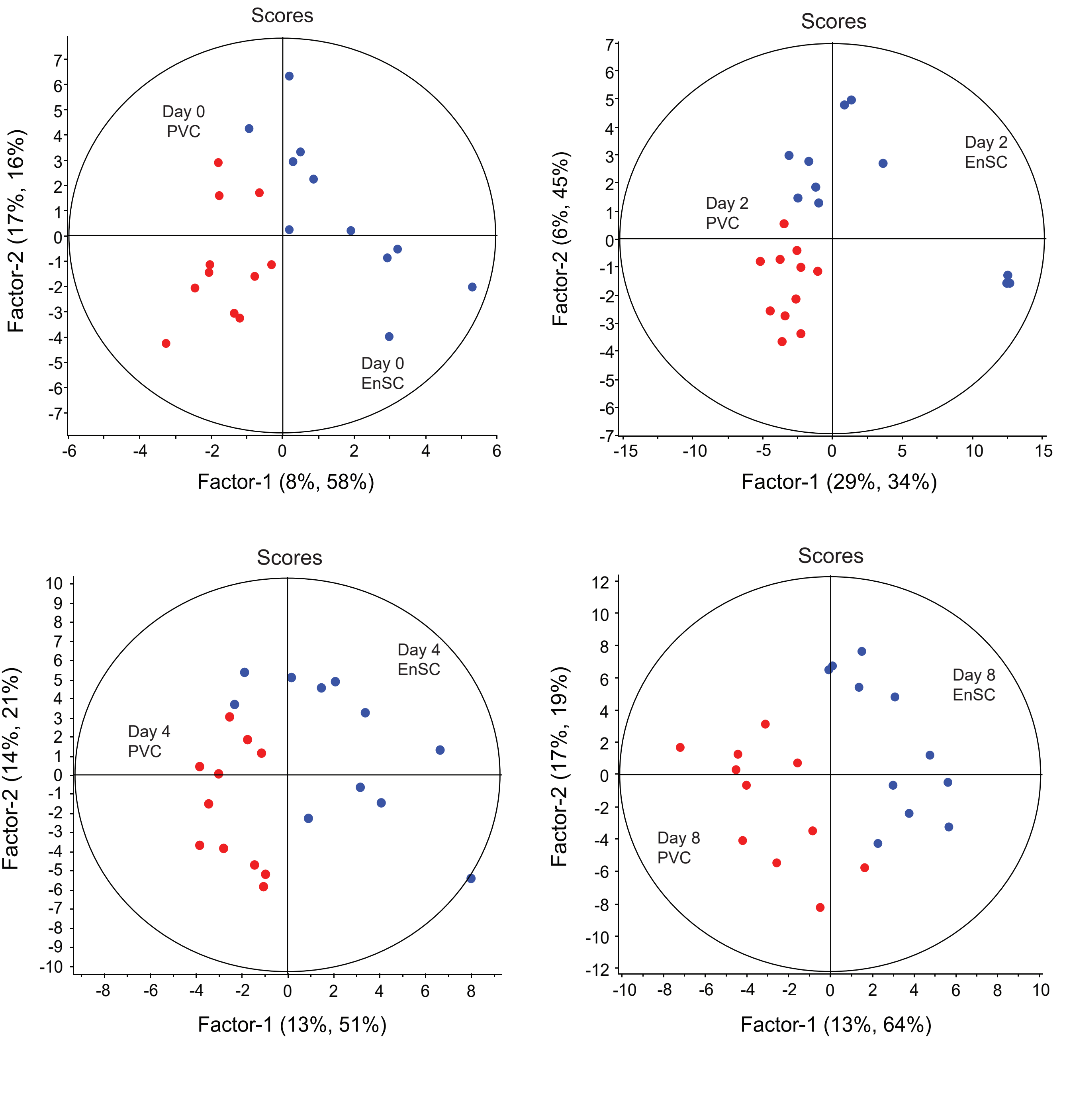

Figure S5 Partial least squares regression analysis separates PVC and EnSC at each decidual time point
