# Supplementary material for: Exometabolomic Analysis of Decidualizing Human Endometrial Stromal and Perivascular Cells
Source: Front Cell Dev Biol. 2021 Jan 28;9:626619. doi: 10.3389/fcell.2021.626619 (PMC7876294; doi:10.3389/fcell.2021.626619)
Supplement: Supplementary file 6 [file Data_Sheet_6.PDF]

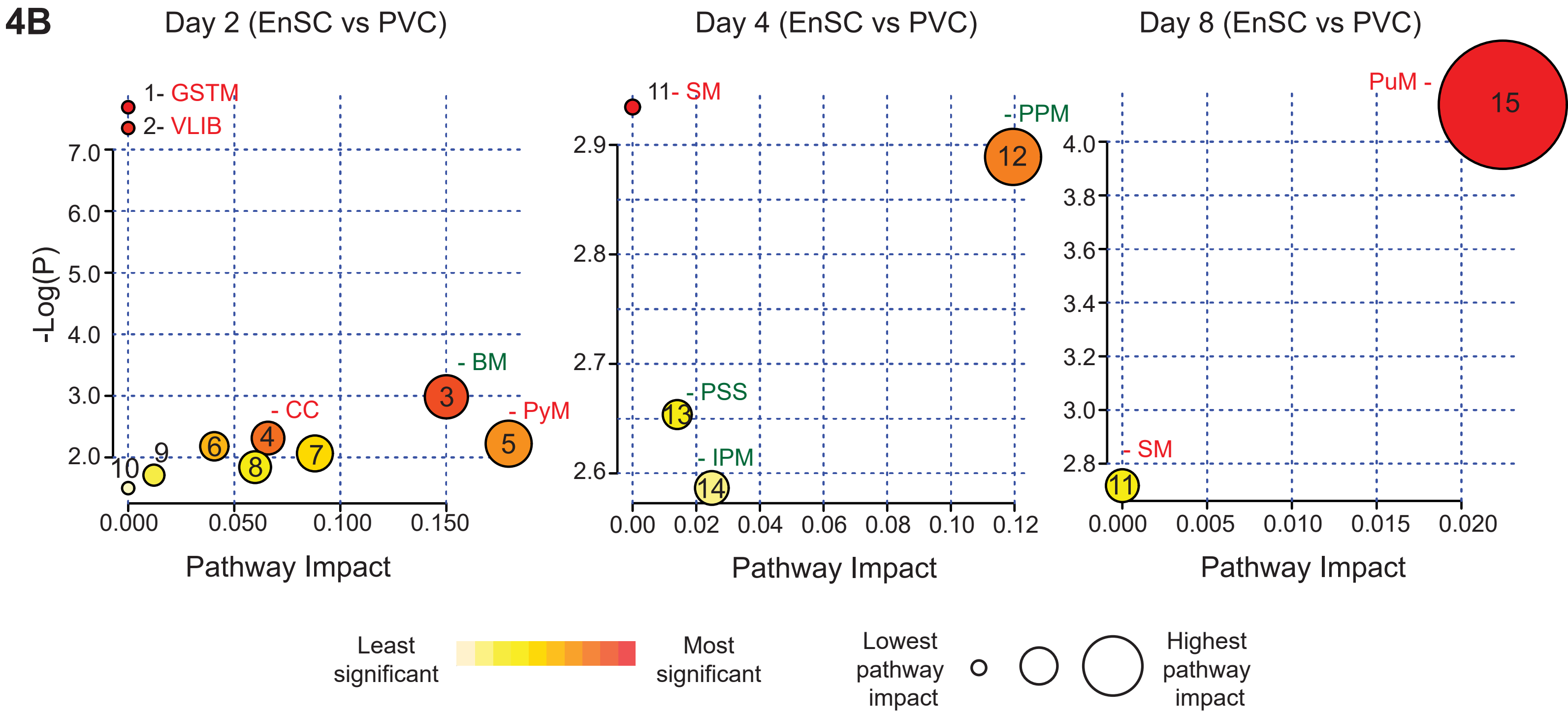

Day 2

Number	Pathway
1	Glycine, serine & threonine metabolism
2	Valine, leucine & isoleucine biosynthesis
3	Biotin metabolism
4	Citrate Cycle (TCA cycle)
5	Pyruvate metabolism
6	Propanoate metabolism
7	Glycolysis/ Gluconeogenesis
8	Cysteine and methionine metabolism
9	Arginine and proline metabolism
10	Aminoacyl- tRNA biosynthesis

Day 4

Number	Pathway
11	Sphingolipid metabolism
12	Pentose phosphate metabolism
13	Phosphatidylinositol signalling system
14	Inositol phosphate metabolism

Day 8

Number	Pathway
11	Sphingolipid metabolism
15	Pentose phosphate metabolism

Key
Lower levels in PVC
Higher levels in PVC

Differential pathways corresponds to Figure 4B

Figure S6: Metaboanalyst Pathway analysis comparing PVC to EnSC of untargeted metabolomics by for day two, four and eight decidualization. Pathways labelled: glycine, serine, and threonine metabolism (GSTM), valine, leucine, and isoleucine biosynthesis (VLIB), biotin metabolism (BM), tricarboxylic acid cycle (TCA), pyruvate metabolism (PyM), sphingolipid metabolism (SM),pentose phosphate metabolism (PPM), phosphatidylinositol signalling system (PSS), inositol phosphate metabolism (IPM) and purine metabolism (PuM). Other pathways, are labelled with numbers and can be found in the table.
