# Supplementary material for: Exometabolomic Analysis of Decidualizing Human Endometrial Stromal and Perivascular Cells
Source: Front Cell Dev Biol. 2021 Jan 28;9:626619. doi: 10.3389/fcell.2021.626619 (PMC7876294; doi:10.3389/fcell.2021.626619)
Supplement: Supplementary file 7 [file Table_1.DOCX]

**Table S1** Patient demographics

| **Sample Number** | **Age** | **BMI** | **Live birth** | **1st Trimester loss** |
| --- | --- | --- | --- | --- |
| 12 | 38 (34.75-39.25) | 24 (23-25) | 0 (0-1) | 4 (2-4.5) |
| All data are median (Interquartile range, Q1-Q3) | | | | |
